# Assessments for Prodromal Alzheimer’s Disease Having Positive Amyloid Pathology Using Plasma Biomarkers And APOE Genotype

**DOI:** 10.1002/alz.089916

**Published:** 2025-01-09

**Authors:** Pei‐Ning Wang, Ming‐Jang Chiu, Marwan N. Sabbagh, Charles S.Y. Yang

**Affiliations:** ^1^ National Yang‐Ming University School of Medicine, Taipei Taiwan; ^2^ National Taiwan University Hospital, College of Medicine, National Taiwan University, Taipei Taiwan; ^3^ Barrow Neurological Institute, Phoenix, AZ USA; ^4^ MagQu Co., Ltd., Taiwan, New Taipei City Taiwan

## Abstract

**Background:**

With the success in developing drugs able to slow cognitive decline in Alzheimer’s disease (AD), the disease managements of AD become aggressive and positive. The target patients of drug are the prodromal AD, i.e. clinical dementia rating being 0.5 and mini‐mental status examination being between 20 and 28, having positive amyloid pathology. In practical, it seems to have big burdens in finding prodromal AD patients have positive amyloid pathology at neurological departments in hospitals. The impact of drug in managing AD is intensively suppressed. A pre‐screening tool for finding target patients is needed. By integrating the reported assessments of prodromal AD and positive amyloid positron emission tomography (PET) using plasma biomarkers, a pre‐screening flow chart is proposed.

**Method:**

A multi‐centered study was done to validate assessments of amnestic mild cognitive impairment (aMCI) and Alzheimer’s disease dementia (ADD) using plasm concentration ratio of amyloid β 1‐42 (Aβ_1‐42_) to Aβ_1‐40_ (Aβ_1‐42_/Aβ_1‐40_) and the concentration product of Aβ_1‐42_ and total tau protein (T‐Tau) (Aβ_1‐42_xT‐Tau), which were assayed with immunomagnetic reduction (IMR). Explorations of pre‐screening model of amyloid pathology in prodromal using plasma Aβ_1‐42_ and APOE genotype in prodromal AD was investigated in the multi‐centered study.

**Result:**

The levels of Aβ_1‐42_/Aβ_1‐40_ or Aβ_1‐42_xT‐Tau in plasma show high accuracy (> 85%) in assessing prodromal AD. The combination of plasma Aβ_1‐42_ and APOE genotypes show the high prediction (> 84%) in positive amyloid PET in prodromal AD. These results demonstrate the feasibilities of assessing prodromal AD using plasma Aβ_1‐42_/Aβ_1‐40_ or Aβ_1‐42_xT‐Tau, followed by combing plasma Aβ_1‐42_ with APOE genotypes to predict the positive amyloid PET.

**Conclusion:**

Such pre‐screening flow chart could breakdown the bottleneck of finding prodromal AD having positive amyloid PET in clinic and promote the impact of drug to the managements of AD.

